# A Chinese family affected by lynch syndrome caused by *MLH1* mutation

**DOI:** 10.1186/s12881-018-0605-x

**Published:** 2018-06-22

**Authors:** Shuqin Jia, Meng Zhang, Yu Sun, Hai Yan, Fangping Zhao, Ziyu Li, Jiafu Ji

**Affiliations:** 1 Center for Molecular Diagnostics, Key laboratory of Carcinogenesis and Translational Research (Ministry of Education), Peking University Cancer Hospital & Institute, No.52 Fucheng Road, Haidian District, Beijing, 100142 China; 2 Department of Pathology, Key laboratory of Carcinogenesis and Translational Research (Ministry of Education), Peking University Cancer Hospital & Institute, Beijing, China; 3 Department of Pathology, Duke University Medical Center, Durham, NC USA; 4 Genetron Health Co., Ltd, Beijing, China; 5 Department of Gastrointestinal Surgery, Key laboratory of Carcinogenesis and Translational Research (Ministry of Education), Peking University Cancer Hospital & Institute, No.52 Fucheng Road, Haidian District, Beijing, 100142 China

**Keywords:** Lynch syndrome, Hereditary, Colorectal carcinoma, Genetic counseling, Case report

## Abstract

**Background:**

Lynch syndrome (LS) is caused by mutations in DNA mismatch repair (MMR) genes, which accounts for 3–5% of colorectal cancer. The risks of several types of cancer are greatly increased among individuals with LS. In this study, 4 members of a Chinese family with a *MLH1* pathogenic variant, resulting in colonic carcinoma, was reported.

**Case presentation:**

A 52-year-old colon cancer female was brought to us with a family history of colon cancer. Genetic counseling traced 4 members in her family with colon cancer (mother and 3 siblings including the proband) as well as other cancer types. Next generation sequencing (NGS) with a multiple gene panel including MMR genes showed a germline mutation in *MLH1* (c.1852_1854delAAG, p.K618del) in all 3 affected family members and confirmed the diagnosis of Lynch syndrome. In addition, this mutation was also identified in a asymptomatic offspring, who was then recommended to a prophylactic measure against cancer. A personalized health care plan was implemented for monitoring the condition and progression of the affected individuals.

**Conclusion:**

Based on public database searching followed by pedigree verification, p.K618del variant in *MLH1* is a pathogenic mutation, which supported the diagnosis of LS. This case highlights the importance of diagnosis and management in patients with hereditary cancer syndromes, particularly for asymptomatic family members.

**Electronic supplementary material:**

The online version of this article (10.1186/s12881-018-0605-x) contains supplementary material, which is available to authorized users.

## Background

Approximately 1.4 million new colorectal cancer (CRC) cases occurred in 2012 worldwide [1]. The majority of CRCs are sporadic which is caused by comprehensive genetic and environmental factors, whereas, hereditary CRCs account for 5–10% of total CRCs. Moreover, an approximately 3 to 5% of CRCs are caused by Lynch syndrome (LS) which is also called hereditary non-polyposis colorectal cancer (HNPCC) [2, 3]. Patients with LS have a 52–82% lifetime risk of colorectal carcinoma and 6–13% risk of gastric cancer, as well as increased risks of developing other cancer types including small bowel, liver, gallbladder ducts, urinary tract, pancreas, brain and skin. In females, LS confers increased risks of both endometrial (25–60%) and ovarian cancers (4–12%) [4]. LS cancer risk is inherited in an autosomal dominant pattern, and is caused by germline variations in DNA mismatch repair (MMR) genes such as *MLH1*, *MSH2*, *MSH6*, or *PMS2*. Approximately 90% of LS patients could potentially carry variations in *MLH1* and *MSH2*, 7% carry mutations in *MSH6*, and less than 5% are likely to have *PMS2* mutations [4]. The normal function of the MMR proteins is to correct errors during DNA synthesis and prevent recombination between non-identical sequences. Mutations in any of these genes may lead to genomic instability, manifesting microsatellite instability (MSI) [5]. These gene variations could be identified by sequencing methods. Since the breakthrough of the next-generation sequencing (NGS) technology, the scale of genomic information acquired by such manner is significantly increased while the cost is dramatically reduced. Its core idea is sequencing by synthesis, which is to identify the DNA sequencing by capturing the signal as a nucleotide added to the growing strand. Microsatellites are short repetitive DNA sequences distributed throughout human genome. They are widely used for DNA profiling in cancer diagnosis. Increases or decreases in repeat length within the microsatellites markers, detected from the test DNA sample and normalized by matching normal, were analyzed by capillary electrophoresis. Alleles that are presented in the test DNA sample but not found in the corresponding normal samples indicate MSI. The development of these technologies enables the diagnosis of hereditary cancer syndrome more efficient and accurate than ever before, providing clinicians with quick guidance on the subsequent health care management strategies.

Cancer genetic counseling is essential for the health management of patients and their families with hereditary cancer syndromes. However, there is still a lack of standards in clinical practice in China. In this study, we report a case of a Chinese family with LS, and how we make the diagnosis and a implementation of a personalized health management plan using modern clinical molecular techniques and professional genetic counseling.

## Case presentation

The patient, a 52-year-old female, was admitted to the department of gastrointestinal surgery of Peking University Cancer Hospital & Institute in September, 2016, due to space-occupying lesions in the colon found by colonoscopy during medical examination 2 weeks before. Histopathological examination of endoscopic biopsy specimens indicated moderately differentiated colonic adenocarcinoma. Contrast-enhanced computer tomography (CT) of her abdomen demonstrated that the intestinal wall was thickened about 14 mm in the transverse colon, and several small lymph nodes (7 mm) scattered around the intestine were detected (Fig. 2a and b). Laboratory examination revealed that the levels of CEA and CA72.4 increased to 15.17 ng/ml and 20.88 U/ml respectively. Laparoscopic examination confirmed the tumor (6 cm × 5 cm) was located in the hepatic flexure of the colon (Fig. 2c and d).

**Fig. 1:**
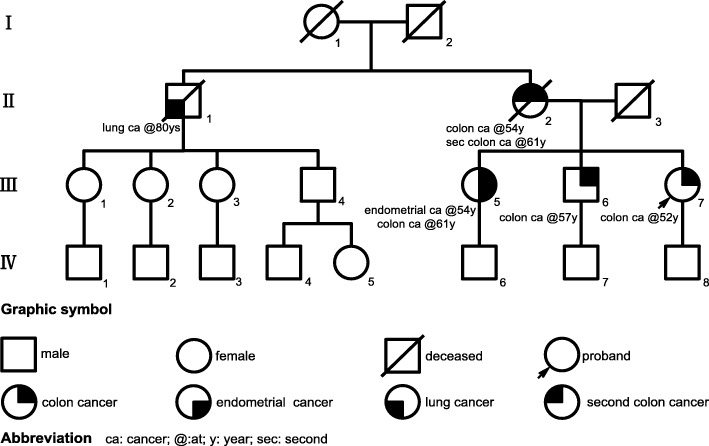
Pedigree. Affected individuals III5, III6 and III7(proband) all had colon cancer and all carry the same *MLH1* mutation (Table 1). With the history of colon cancer, II2 is predicted to the carrier of *MLH1* mutation in the family. Among offspring of the affected individuals, IV8 carries the same *MLH1* mutation (Table 1) and underwent preventative therapy

The patient (III7) had no other major medical history, except a family history of colon cancer in 3 out of 5 first-degree relatives (mother II2; sister, III5; brother, III6). In particular, the patient’s mother (II2) was diagnosed two separate primary colon cancer at the age 54 and 61 at different sites. Her sister (III5) was diagnosed with endometrial cancer and colon cancer at the age of 54 and 61 respectively (Fig. 1). The patient was referred to our cancer genetic counseling clinic for LS genetic testing. Based on Amsterdam II criteria, the proband was diagnosed with LS.

To confirm the diagnosis, all affected individuals (III5, 6 and 7) underwent genetic testing of a 101-gene panel by next generation sequencing. Peripheral blood was collected to extract genomic DNA (gDNA). The gDNA was then used to generate libraries according to the protocols suggested by Illumina. A custom targeted capture kit, covering all exons of the 101 genes, was designed (Agilent Technologies, Additional file 1: Table S1) [6]. Qualified libraries were subsequently sequenced on the Illumina HiSeq 2500 platform with 2 × 150 bp configuration. Reads were aligned to the reference human genome GRCh37 with BWA and PCR duplications were marked using Picard tools (version 1.57). To further increase the specificity for mutation calling, realignment and base recalibration were conducted using Genome Analysis tool kit (GATK). All samples were tested at least in an average depth of 200-fold coverage. Bases with a minimum of 30-fold coverage was required at every targeted position (Additional file 2: Table S2). The missense, nonsense, indel and splice site mutations that located at the upstream or downstream 1-2 bp of exon, whose frequency are below 5% in at least one pubic population database were retained (Additional file 3: Table S3). According to the American College of Medical Genetics (ACMG) standards and guidelines for the interpretation of sequence variants, all the gene variants were classified into 5 grades. Therefore, 14 mutations at least carried by two first-degree relatives were listed. A pathogenic variant (class 5) in *MLH1* (c.1852_1854delAAG, p.K618del) was identified in all patient’s blood samples (Table 1).

**Table 1:** Sequencing result on genes associated with hereditary cancer syndrome in family members

Gene	cDNA change	AA change	Patient						Clinical
			III5	III6	III7	IV 6	IV 7	IV 8	significance
*EPCAM*	c.298G > A	p.D100N	het	wt	het	het	wt	het	Likely benign
*FANCE*	c.1028G > A	p.R343Q	het	het	wt	het	het	wt	Benign
*MC1R*	c.488G > A	p.R163Q	het	wt	het	wt	het	hom	Benign
*MLH1*	c.1852_1854delAAG	p.K618del	het	het	het	wt	wt	het	Pathogenic
*MLH1*	c.655A > G	p.I219V	het	het	het	het	wt	wt	Benign
*NBN*	c.1690G > A	p.E564K	het	het	het	wt	wt	het	Benign
*PALLD*	c.2069C > A	p.P690H	wt	het	wt	wt	het	wt	Benign
*PMS2*	c.379G > A	p.A127T	wt	het	het	wt	het	wt	Likely benign
*PTCH1*	c.3583A > T	p.T1195S	het	het	het	wt	het	wt	Benign
*RHBDF2*	c.28A > C	p.S10R	het	het	het	wt	het	wt	Benign
*SDHA*	c.113A > T	p.D38V	wt	wt	het	wt	wt	het	Likely benign
*SLX4*	c.1231C > T	p.R411W	het	het	het	het	het	wt	Likely benign
*TSC2*	c.856A > G	p.M286 V	het	het	het	wt	wt	het	Benign
*TSC2*	c.500G > T	p.W167 L	het	het	het	wt	wt	het	VUS

Next generation sequencing was performed using 101-gene panel developed by Genetron Health (Beijing) Co., Ltd

*AA* amino acid, *Wt* wild type, *Het* heterozygous mutation, *Hom* homozygous mutation, *VUS* variants of uncertain significance

**Fig. 2:**
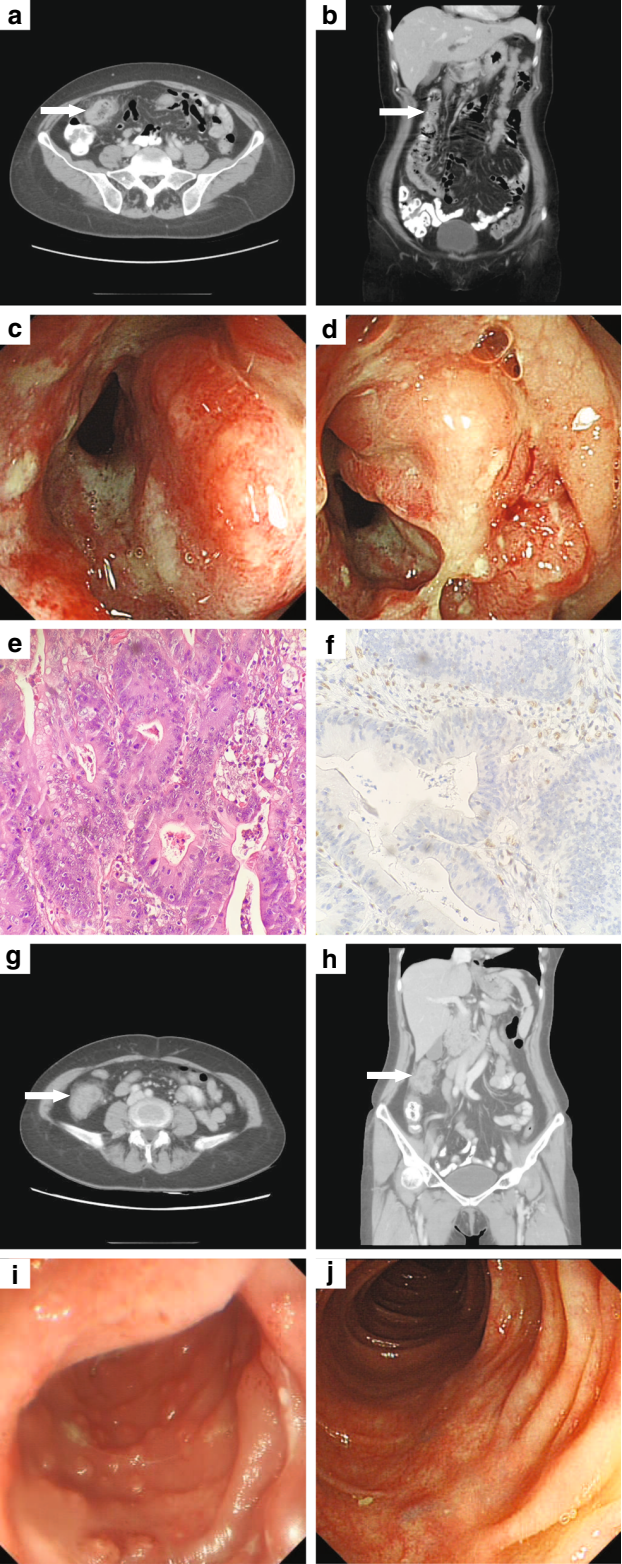
Clinical examination results. **a** and **b** Abdominal computed tomography (CT) scan of the proband (III7) revealed an obstructing mass located in the hepatic flexure of the colon (arrow). a, axial and b, coronal. **c** and **d** A space-occupying lesion in colon by colonoscopy in III7. **e** HE staining demonstrates moderately-differentiated colon adenocarcinoma with glandular architecture, nuclear atypia and dysregulated cell proliferation. **f** immunochemistry stain of MLH1. **g** and **h** Abdominal computer tomography of III5 showed a obstructing mass in the same location of colon as the proband (III7) (arrow). **g**, axial view. **h**, coronal view. **i** and **j** Colonoscopy of IV8. **i** Before the preventative treatment, ileocecal mucosa showed sign of chronic inflammation with erosion, lymphoid hyperplasia and mild atypical hyperplasia of glandular epithelium. **j** After oral administration of aspirin for 6 months, same site of ileocecal mucosa showed improved appearance overall

Laparoscopy-assisted colectomy was performed on the proband to resect right colonic mass and its surrounding tissue followed by ileocolonic anastomosis. In addition, the clinicopathologic stage was pT3N0M0 and no complications occurred in the perioperative period. Conventional hematoxylin and eosin staining and immunohistochemistry were performed on resected specimens to confirm the malignancy (Fig. 2e and f). Additionally, abdominal CT of III5 showed a obstructing mass in the same location of colon as the proband (III7) (Fig. 2g and h). Moreover, according to the medical record for the proband’s brother (III6) in another hospital, a tumor was found in his hepatic flexure of colon. Immunohistochemistry results showed MMR deficiency in all tumor tissues of the 3 cancer patients (Table 2). Subsequently, MSI testing was performed using MSI Analysis System Version 1.2 (Promega). Tumor DNA was extract from formalin fixed paraffin embedded sections. Genomic DNA extracted from white blood cell was used as normal control. Seven markers were amplified using fluorescent PCR. The PCR products were separated by capillary electrophoresis using Applied Biosystem 3130 Genetic Analyzer. GeneMapper Analysis Software was used to analyze the output data. The MSI results indicated that the 3 siblings with cancer (III5, 6 and 7) were all microsatellite instability-high (MSI-H) (Table 2). Based on the results of MSI and the proband’s clinical stage, no adjuvant chemotherapy was given after surgery.

**Table 2:** Detection of microsatellite instability

Patient		Proband III7	Proband’s Sister III5	Proband’s Brother III6
MSI status		MSI-H	MSI-H	MSI-H
Genetic testing for microsatellite instability	BAT-25	MSI	MSI	MSI
	BAT-26	MSS	MSI	MSI
	NR-21	MSI	MSI	MSI
	NR-24	MSI	MSI	MSI
	Mono-27	MSS	MSI	MSI
Immunohistochemistry of MMR proteins	MLH1	–	–	–
	MSH2	+	+	–
	MSH6	+	+	–
	PMS2	–	–	+

Microsatellite instability was accessed at both protein and genetic levels. Immunohistochemistry of MMR proteins (MLH1, MSH2, MSH6 and PMS2) was performed to demonstrate the deficiency of MMR system in affected individuals. “-” suggests the deficiency of corresponding proteins and “+” suggests a normal expression of corresponding proteins. Deficiency in MMRs results in MSI

Genetic screening for MSI was performed using the BAT-25, BAT-26, NR-21, NR-25, and Mono-27 markers. *MSS* microsatellite stable, *MSI* microsatellite instability, when none of the five markers showed instability. *MSI-H* MSI-high, when two or more of the five markers showed instability. *MSI-L* MSI-low, when only one of the five markers showed instability

To screen and evaluate the cancer developing risk in the offspring, children of affected individuals were also enrolled for genetic testing. And 1 (IV8) out of 3 carries the same *MLH1* mutation to the proband. However, this carrier has no symptoms or confirmed diagnosis of cancer. Endoscopy was performed on IV8 for further examination and the ileocecal mucosa showed signs of dysplasia, including chronic inflammation with erosion, lymphoid hyperplasia and mild atypical hyperplasia of glandular epithelium (Fig. 2i). The asymptomatic individual was given oral administration of aspirin as a preventative treatment, and 6-months follow-up showed improved appearance with colonoscopy examination (Fig. 2j). A healthcare plan was proposed to this offspring including colonoscopy and urine test once a year, and gastroscopy every 3~ 5 years after 35 years old. All individuals carrying the *MLH1* mutation in this family will be monitored on a long term basis.

The CARE guidelines were followed in reporting this case.

## Discussion and conclusions

CRC is the fifth diagnosed cancer in China [7]. The estimated numbers of CRC related new cases and deaths in China in 2015 were about 376,300 and 191,000 respectively. Among these patients, 75% was sporadic, whereas 25% represented a family history of cancer. Additionally, inherited colorectal cancer accounts for 5–10% of total CRCs, including LS, familial adenomatous polyposis, MUTYH-associated polyposis, Li-Fraumeni syndrome, Peutz-Jeghers syndrome and Juvenile polyposis syndrome [8]. LS is considered as an assemblage of related cancers characterized by defects in DNA MMR genes including *MLH1*, *MSH2*, *MSH6*, and *PMS2*. It is estimated that 90% of LS related CRCs and less than 10% of sporadic CRCs show MSI. LS confers a 52–82% lifetime risk of CRCs. Individuals carrying *MLH1* or *MSH2* pathogenic variations appear to have the highest risk developing CRC, which occurs at the age of 44 on average.

In this case, a typical Chinese LS family was reported. With thorough genetic counseling prior to genetic testing, a clear LS history was established: the proband’s mother (II2) suffered from second primary colon cancer, and her sister (III5) was also affected by endometrial cancer. Although the family history indicated the clinical diagnosis of LS, we could not make a definite diagnosis of LS without genetic testing. Therefore, a 101-gene panel that covered 65 common hereditary cancer syndromes was used. From clinical perspective, a single MMR gene testing once at a time using Sanger sequencing is more likely to be appropriate for the proband and her relatives. However, in this study, the proband was considered as LS based on family history without further test prior to the genetic test. The results of IHC and MSI were obtained after the surgery when the diagnosis was confirmed. Considering the comparable cost of Sanger sequencing all exons of different MMR genes and the NGS sequencing, we preferred NGS for its faster turnaround time. Besides, we would like to explore if there is any other potential colorectal cancer predisposing genes in Chinese population. Genetic testing reveals all 3 affected siblings carrying a pathogenic variant in *MLH1* (c.1852_1854delAAG). In addition, their tumors developed in the common location in colon. Further studies may help understand the pathological impact of this mutation during tumorigenesis.

Meanwhile, all offspring at risk of developing cancer were recommended for genetic testing and results showed that the proband’s son (IV8) inherited the pathogenic variation. A chemoprevention strategy of aspirin oral administration was prescribed based on preclinical studies and clinical trials [9–11]. Furthermore, a personalized healthcare management plan was recommended to the young carrier for regular surveillance. As of this study submission, signs of colonic dysplasia were under control after 6 months of aspirin treatment.

MSI-H caused by germline mutations in *MLH1* is not only a key component of the pathogenesis of CRC but also act as a positive prognostic maker. Moreover, it can be used to predict the efficacy of chemotherapy [12] and immunotherapy [13]. All affected cancer patients in the family that were diagnosed with cancer underwent surgery without adjuvant therapies based on NCCN (V2.2016) guidelines [14]. In the case of the proband (III7), the latest CT scan was performed 16 months after the surgery and did not show any sign of cancer recurrence, and the surveillance and follow-up of this LS family is still ongoing.

The standard procedure of cancer genetic counseling includes pre-test counseling, result disclosure and follow-up. The purpose of genetic counseling is to help at-risk individuals and their families to understand the risks of cancers, to disclose the genetic test results, to discuss options for risk management and family planning, and to provide psychosocial support for individuals as needed. Cancer genetic counseling is just sprouting in China and will be facing many challenges such as shortage of qualified genetic counselors. This is expected to be solved with a better understanding of cancer genetics in both cancer patients and medical practitioners as well as better preventative education to the general population.

## Additional files

Additional file 1: **Table S1.** 101 gene panel. The gene list of targeted capture panel used in the case. (XLSX 11 kb)

Additional file 2: **Table S2.** All six family members gene test result. All six family members gene test results displayed in order of gene. (XLSX 770 kb)

Additional file 3: **Table S3.** Individual sequencing result. All six family members gene test results displayed in individual form. (XLSX 32 kb)

## Abbreviations

ACMG: American College of Medical Genetics
CRC: Colorectal cancer
CT: Computer tomography
GATK: Genome analysis tool kit
gDNA: Genomic DNA
HNPCC: Hereditary non-polyposis colorectal cancer
LS: Lynch syndrome
MMR: DNA mismatch repair
MSI: Microsatellite instability
MSI-H: Microsatellite instability-high
NGS: Next-generation sequencing
